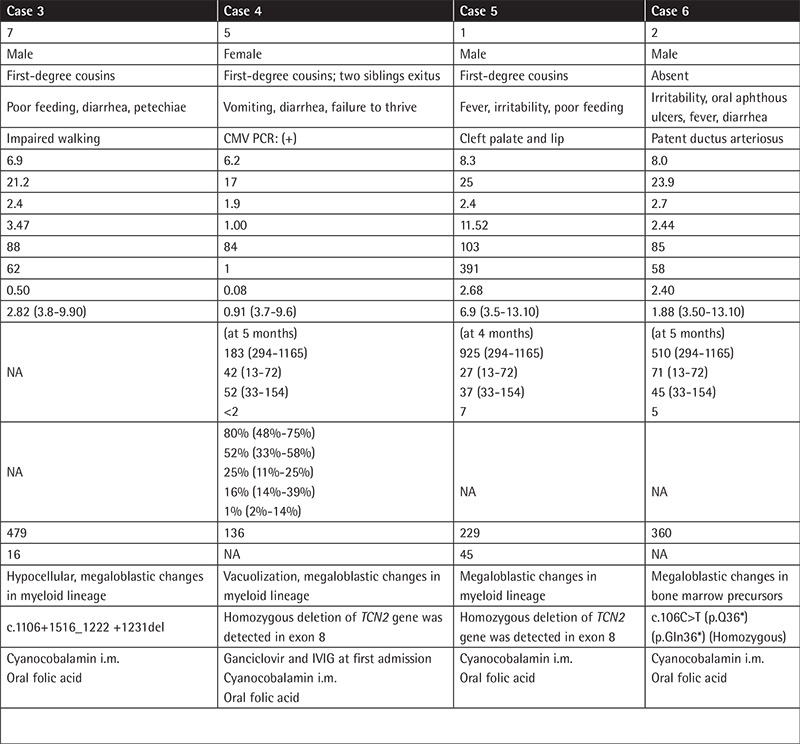# Different Presentations of Patients with Transcobalamin II Deficiency: A Single-Center Experience from Turkey

**DOI:** 10.4274/tjh.galenos.2018.2018.0230

**Published:** 2019-02-07

**Authors:** Selma Ünal, Feryal Karahan, Tuğba Arıkoğlu, Asuman Akar, Semanur Kuyucu

**Affiliations:** 1 Mersin University Faculty of Medicine, Department of Pediatric Hematology, Mersin, Turkey; 2 Mersin University Faculty of Medicine, Department of Pediatric Allergy and Immunology, Mersin, Turkey; 3 Mersin University Faculty of Medicine, Department of Pediatric Infectious Diseases, Mersin, Turkey

**Keywords:** Transcobalamin II, Vacuolization, Immune deficiency, Neurological impairment, Hemophagocytic lymphohistiocytosis

## Abstract

**Objective:**

Transcobalamin II deficiency is a rare autosomal recessive disease characterized by decreased cobalamin availability, which in turn causes accumulation of homocysteine and methylmalonic acid. The presenting clinical features are failure to thrive, diarrhea, megaloblastic anemia, pancytopenia, neurologic abnormalities, and also recurrent infections due to immune abnormalities in early infancy.

**Materials and Methods:**

Here, we report the clinical and laboratory features of six children with transcobalamin II deficiency who were all molecularly confirmed.

**Results:**

The patients were admitted between 1 and 7 months of age with anemia or pancytopenia. Unexpectedly, one patient had a serum vitamin B12 level lower than the normal range and another one had nonsignificantly elevated serum homocysteine levels. Four patients had lymphopenia, four had neutropenia and three also had hypogammaglobulinemia. Suggesting the consideration of transcobalamin II deficiency in the differential diagnosis of immune deficiency. Hemophagocytic lymphohistiocytosis was also detected in one patient. Furthermore, two patients had vacuolization in the myeloid lineage in bone marrow aspiration, which may be an additional finding of transcobalamin II deficiency. The hematological abnormalities in all patients resolved after parenteral cobalamin treatment. In follow-up, two patients showed neurological impairments such as impaired speech and walking. Among our six patients who were all molecularly confirmed, two had the mutation that was reported in transcobalamin II-deficient patients of Turkish ancestry. Also, a novel *TCN2* gene mutation was detected in one of the remaining patients.

**Conclusion:**

Transcobalamin II deficiency should be considered in the differential diagnosis of infants with immunological abnormalities as well as cytopenia and neurological dysfunction. Early recognition of this rare condition and initiation of adequate treatment is critical for control of the disease and better prognosis.

## Abstract (tr)

**Amaç:**

Transkobalamin II eksikliği homosistein ve metilmalonik asit birikimine neden olan kobalamin yararlanımının azaldığı nadir bir otozomal resesif hastalıktır. Erken dönemde gelişme geriliği, diyare, megaloblastik anemi, pansitopeni, nörolojik anormallikler ve ayrıca immün bozukluklara bağlı tekrarlayan enfeksiyonlarla karekterizedir.

**Gereç ve Yöntemler:**

Bu çalışmada, transkobalamin II eksikliği tanısı moleküler olarak konfirme edilen altı hastanın klinik ve laboratuvar özellikleri sunulmuştur.

**Bulgular:**

Hastalar 1-7 ay arasında anemi veya pansitopeni ile başvurdu. Beklenmedik şekilde, bir hastanın serum vitamin B12 düzeyi normalden düşüktü ve diğer bir hastanın serum homosistein düzeyi belirgin düzeyde yüksek değildi. Dört hastada lenfopeni, dört hastada nötropeni ve üç hastada ayrıca hipogamaglobulinemi olup bu bulgular immün yetersizliğin ayırıcı tanısında transkobalamin II eksikliğinin düşünülmesi gerektiğini göstermektedir. Bir hastada hemofagositik lenfohistiyositoz saptandı. Ayrıca, iki hastanın kemik iliği aspirasyonunda myeloid seride vakuolizasyon olup transkobalamin II eksikliğinin ek bir bulgusu olabilir. Parenteral kobalamin tedavisinden sonra tüm hastaların hematolojik anormallikleri düzeldi. Takipte iki hastada konuşmada ve yürümede bozulma şeklinde nörolojik anormallikler saptandı. Tümü moleküler olarak doğrulanmış altı olgumuzdan ikisinde, transkobalamin II eksikliği olan Türk ırkına ait hastalarda bildirilen mutasyon mevcuttu. Ayrıca, kalan olgulardan birinde yeni bir *TCN2* gen mutasyonu tespit edildi.

**Sonuç:**

Transkobalamin II eksikliği sitopeni ve nörolojik bozuklukların olduğu gibi immünolojik anormalliklerin de olduğu infantlarda ayırıcı tanıda düşünülmelidir. Bu nadir hastalığın erken tanınması ve uygun tedavinin başlanması hastalığın kontrolü ve daha iyi bir prognoz açısından kritiktir.

## Introduction

Transcobalamin II (TC) deficiency is a rare autosomal recessive disease in early infancy caused by mutations in the *TCN2* gene [1]. TC is a transport protein for vitamin B12 and facilitates its cellular uptake by receptor-mediated endocytosis [1]. A deficiency of TC results in a lack of vitamin B12 entry into cells, leading to intracellular cobalamin depletion [2]. The presenting clinical features are failure to thrive, diarrhea, pancytopenia, neurologic abnormalities, and infections due to immunodeficiency [3,4,5,6].

The diagnosis is suspected based on the presence of clinical and laboratory features [5]. Patients with TC deficiency have elevated homocysteine and methylmalonic acid levels [2,7]. Treatment with parenteral vitamin B12 is highly effective on clinical and biological signs. It is important to establish the diagnosis of TC deficiency as early as possible because with a significant delay in diagnosis, and therefore treatment, the neurological abnormalities can become irreversible [8]. 

In the present study, we report the clinical and laboratory features of six children diagnosed with TC deficiency in our hospital.

## Materials and Methods

We retrospectively reviewed the medical records of six children with a diagnosis of TC deficiency in our hospital. Complete blood count, serum vitamin B12, folic acid, homocysteine and immunoglobulin levels, percentage of lymphocyte subsets, bone marrow aspiration, and molecular analysis results were obtained from medical charts. Treatment regimens given to the patients were recorded. The clinical and laboratory findings of our patients are shown in Table 1-Continued.

## Results

### Case 1

A 2-month-old male baby was admitted with complaints of fever, cough, diarrhea, and respiratory distress. The parents were first-degree cousins. The constellation of clinical features such as prolonged fever and splenomegaly and laboratory findings (cytopenia in peripheral blood, elevated ferritin, triglyceride and liver enzymes, and hemophagocytosis in the bone marrow) suggested the diagnosis of hemophagocytic lymphohistiocytosis (HLH). Cytomegalovirus (CMV) PCR was found to be positive and he was given ganciclovir therapy. Intravenous immunoglobulin was added to the therapy due to the presence of hypogammaglobulinemia. Percentages of lymphocyte subsets were in the normal ranges. A second bone marrow aspiration demonstrated megaloblastic changes in the erythroid series. The patient’s serum vitamin B12 level was normal; however, the serum homocysteine level (23 µmol/L) was significantly higher than normal. A genetic deficiency of TC was suspected and a homozygous *TCN2* gene mutation was detected in molecular analysis. This 5304-bp deletion began 1516 bp into intron 7 and ended 1231 bp into intron 8. The deletion included all of exon 8 and caused a frameshift to produce a premature stop four codons into the new reading frame. The patient was treated with intramuscular vitamin B12, which was followed by improvement in both clinical and laboratory findings. This case was published as a case report in the literature [9].

### Case 2

A 6-month-old girl presented with complaints of failure to thrive, vomiting, and diarrhea. A diagnosis of sepsis or metabolic disease was suspected and antibiotic therapy was started empirically. She had anemia, neutropenia, and thrombocytopenia. Serum vitamin B12 level was found to be normal; however, serum homocysteine was 53 µmol/L. Megaloblastic changes and vacuolization were prominent in the myeloid lineage in the bone marrow aspiration. Immunological evaluation revealed hypogammaglobulinemia. Percentages of lymphocyte subsets were in the normal range. A genetic deficiency of TC was suspected. The molecular analysis revealed c.1106+1516_1222+1231del mutation. This mutation is not listed in the Human Gene Mutation Database (Cardiff). It is a 5304-bp deletion that begins 1516 bp into intron 7 and ends 1231 bp into intron 8. The deletion includes all of exon 8 and causes a frameshift to produce a premature stop four codons into the new reading frame. The patient was treated with intramuscular vitamin B12 and oral folic acid, which was followed by improvement in hematological response, but a speech deficit was detected at 2 years of age in follow-up.

### Case 3

A 7-month-old boy presented with complaints of poor feeding, diarrhea, and petechiae. He was the child of first-degree cousins. He had pancytopenia. Serum vitamin B12 level was found to be normal and serum homocysteine level was high at the borderline (16 µmol/L). Bone marrow was hypocellular and megaloblastic changes were prominent in the myeloid lineage. A genetic deficiency of TC was suspected. The molecular analysis revealed c.1106+1516_1222+1231del mutation. This mutation is not listed in the Human Gene Mutation Database (Cardiff). It is a 5304-bp deletion that begins 1516 bp into intron 7 and ends 1231 bp into intron 8. The deletion includes all of exon 8 and causes a frameshift to produce a premature stop four codons into the new reading frame. The patient was treated with intramuscular vitamin B12, which was followed by improvement in hematological response, but a delay in walking was detected at 2 years of age in follow-up.

### Case 4

A 5-month-old girl was admitted with failure to thrive, poor feeding, vomiting, and diarrhea. She was the child of first-degree cousins and had a history of sibling death. Laboratory evaluation showed pancytopenia, which required transfusions, and lymphopenia and hypogammaglobulinemia. Percentages of lymphocyte subsets were in the normal range. Serum vitamin B12 level was low (136 pg/mL) and serum homocysteine level could not be measured. CMV PCR was found to be positive. Severe combined immunodeficiency was suspected. Intravenous immunoglobulin, ganciclovir treatment, and antibacterial and antifungal prophylaxis were given. However, bone marrow aspiration showed prominent vacuolization in the myeloid lineage, which suggested Pearson syndrome, and prominent megaloblastic changes in the myeloid lineage. However, molecular analysis did not support the diagnosis of Pearson syndrome. A genetic deficiency of TC was suspected. The patient was treated with intramuscular vitamin B12 and oral folic acid with clinical and hematological improvement. After her family discontinued vitamin B12 therapy, she showed relapse with severe pancytopenia. Vitamin B12 treatment was restarted. The molecular analysis revealed a homozygous *TCN2* gene mutation.

### Case 5

A 1-month-old male baby presented with irritability, fever, and poor feeding. He had a cleft palate and lip. He was the child of first-degree cousins. A diagnosis of sepsis was suspected and antibiotic therapy was started empirically. Complete blood count revealed macrocytic anemia, which required transfusions in follow-up. Serum vitamin B12 and folic acid levels were found to be normal. Bone marrow aspiration showed megaloblastic changes in the myeloid lineage. Serum homocysteine level was 45 µmol/L. A genetic deficiency of TC was suspected. Homozygous deletion of the *TCN2* gene was detected in exon 8. The patient was treated with intramuscular vitamin B12, which was followed by clinical and hematological response.

### Case 6

A 2-month-old male baby presented with complaints of irritability, fever, oral aphthous ulcers, and diarrhea. Patent ductus arteriosus was found in echocardiography. Laboratory evaluation revealed pancytopenia. Serum vitamin B12 and folic acid levels were found to be normal. Serum homocysteine level could not be measured. Bone marrow aspiration was remarkable for megaloblastic changes in erythroid and myeloid cell precursors. A genetic deficiency of TC was suspected. The molecular analysis revealed a homozygous *TCN2* gene mutation: c.106C>T (p.Q36*) (p.Gln36*). The patient was treated with intramuscular vitamin B12 and oral folic acid. He has been asymptomatic in follow-up.

## Discussion

Here, we report six patients with the diagnosis of TC deficiency in our institution. The present study extends our understanding of this rare disease and provides additional data for the clinical and laboratory manifestations. The presenting clinical features are macrocytic anemia, pancytopenia, failure to thrive, gastrointestinal symptoms, and neurologic dysfunction [4,5,10]. In addition to the aforementioned clinical features, recurrent infections can be seen in TC deficiency due to immunological abnormalities. Specific immune abnormalities include hypogammaglobulinemia, specific antibody deficiency, neutropenia, and low T and/or B cell counts [3,5,11]. Consistent with these data, four patients had lymphopenia, four had neutropenia, and three had hypogammaglobulinemia in the present study, suggesting the consideration of TC deficiency in the differential diagnosis of immune deficiency.

HLH is a syndrome characterized by uncontrolled immune response with hyperinflammation [12,13]. Acquired HLH can develop as a result of infections, malignancy, and autoimmunity. In addition, HLH is associated rarely with congenital metabolic disorders [13,14]. Case 1 was reported by Unal et al. [9] in the literature as the first case of TC deficiency presenting with HLH. Defects in cobalamin metabolism were suggested to lead to HLH due to defects in DNA synthesis, secondary NK cell functions, and immune regulation [15]. Hypogammaglobulinemia, lymphopenia, and neutrophil dysfunction have been reported in TC deficiency [3,5,11]. Therefore, it can be considered that immune changes caused by a lack of TC in our patient led to the development of secondary HLH. Potential causative mechanisms of HLH induced by defects of cobalamin metabolism merit further investigation. 

Despite early-onset cobalamin treatment and close monitoring of hematological parameters, patients with TC deficiency have been reported to show neurological deterioration including intellectual disability, attention deficits, tremor, myoclonus, ataxia, delay in language, and motor skills. Epilepsy responsive to antiepileptic medication was also reported [4,5]. The most common neurological complication in the literature was speech deficit [5]. Consistent with the literature, Case 2 had speech deficit and Case 3 had impairment in walking despite intramuscular cobalamin treatment. The starting age and mode of cobalamin treatment influence neurological outcome [4,5]. Patients who are treated early have had a better outcome than those inadequately treated. Nevertheless, the natural course of the disease over time might also result in late-onset neurological symptoms [4,5,8]. We initiated treatment with 1 mg of intramuscular cyanocobalamin daily in the first week and the therapy was continued with 1 mg of cyanocobalamin every second day in the second week. In follow-up, weekly injections of 1 mg of cyanocobalamin were administered based on the clinical and hematological response. In association with cyanocobalamin therapy, folic acid supplementation was also given.

To date, serum vitamin B12 levels have been reported to be normal in cases of TC deficiency [4,10,16]. This is due to the fact that the majority of vitamin B12 in circulation is bound to haptocorrin rather than transcobalamin. As a result, patients with TC deficiency do not show a low level of circulating vitamin B12 [1,2]. However, Schiff et al. [8] reported TC-deficient patients with low vitamin B12 levels. In the present study, Case 4 had the unusual feature of a low serum cobalamin level, similar to the cases reported in Schiff et al.’s [8] study. Homocysteine and methylmalonic acid levels are the metabolic markers of vitamin B12 deficiency [7]. The diagnosis of TC deficiency is confirmed by the measurement of these metabolic markers, although they can rarely be within normal limits [10]. Consistent with these data, the homocysteine levels of Case 3 were in the upper limit of normal. Therefore, our findings indicate that a normal or low serum vitamin B12 level or a slightly elevated homocysteine level does not exclude TC deficiency. Thus, molecular analysis should be done in order to establish a firm diagnosis in subjects with suggested TC deficiency but with normal plasma homocysteine levels.

Two previous studies reported vacuolization in the bone marrow aspiration of TC-deficient patients [10,17]. Similar to those previous studies, vacuolization in the myeloid lineage was seen in the bone marrow aspirations of Cases 2 and 4 in the current study and Pearson syndrome was suspected. Vacuolization is an important finding in Pearson syndrome, which may be related to defects in mitochondrial DNA synthesis [18]. However, it may also be an additional finding in TC deficiency, resulting from a defect of cobalamin metabolism.

Trakadis et al. [5] reported a series of 30 patients with TC deficiency. Molecular testing was performed for 20 families and 17 different mutations were identified. Sixteen families had homozygous and four had compound heterozygous mutations. Different intragenic deletions were identified in 15 of the 20 families. Four mutations resulted in exon skipping [19]: two involved exon 7 and one involved exon 4, while the fourth was a large deletion involving exons 1 to 7. Trakadis et al. [5] reported that a founder mutation may be present in patients of Turkish ancestry, as all presumably unrelated probands of Turkish ancestry shared the same homozygous mutation (c.1106+1516_1222+1231del). This common mutation among the Turkish population was also detected in our Cases 2 and 3, who were living in the same town. In this mutation, the deletion includes all of exon 8 and causes a frameshift to produce a premature stop four codons into the new reading frame. The other mutation in Case 6 (c.106C>T (p.Q36*) (p.Gln36*) produces a premature stop four codons into the new reading frame.

## Conclusion

TC deficiency often presents early in life with multisystem involvement. Therefore, it should be considered in differential diagnosis of infants with cytopenia and neurological dysfunction as well as immunological abnormalities. Early recognition of this rare disease and initiation of adequate treatment is critical for disease control and better prognosis.

## Figures and Tables

**Table 1:**
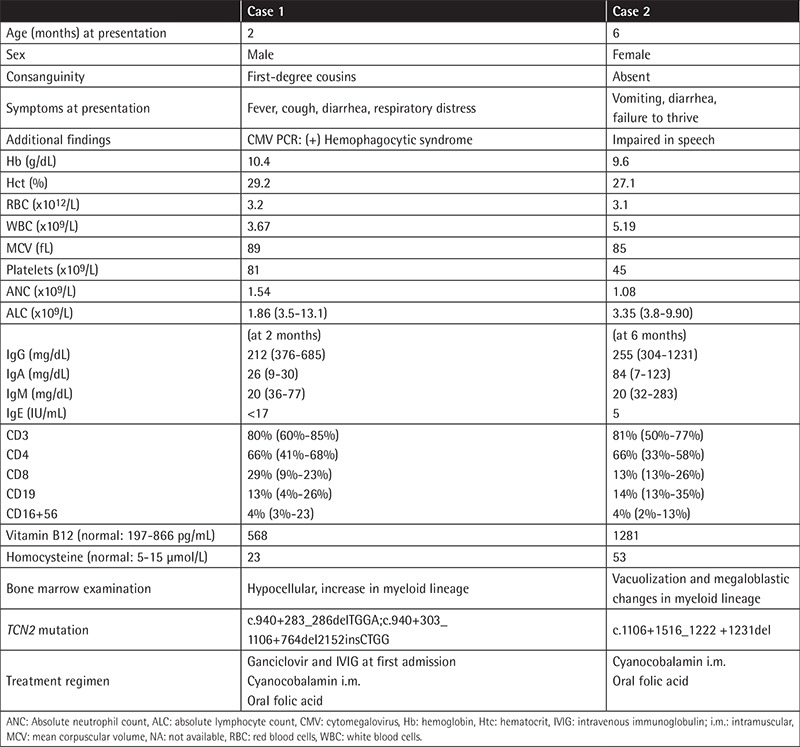
Clinical manifestations and laboratory findings of the patients.

**Table 1. Continued:**